# Usability and Acceptance of Non-Functional Wearable Prototypes for Maternal Health: A Parallel-Group Pilot Study

**DOI:** 10.3390/healthcare14050618

**Published:** 2026-02-28

**Authors:** Julia Jockusch, Sophie Schneider, Andrea Hochuli, Flurin Stauffer, Heike Bördgen, Vanessa Hoop, Marianne Simone Joerger-Messerli, Daniel Surbek, Anda-Petronela Radan

**Affiliations:** 1 Department of Obstetrics and Gynecology, University Hospital Bern, University of Bern, 3010 Bern, Switzerland; 2 Department of Prosthetic Dentistry and Senior Dentistry, Brandenburg Medical School (Theodor Fontane), 14776 Brandenburg an der Havel, Germany; 3 Nanoleq AG, Hofwisenstrasse 50A, 8153 Rumlang, Switzerland; 4 Department for BioMedical Research (DBMR), University of Bern, 3008 Bern, Switzerland

**Keywords:** wearable, pregnancy, usability testing, comfort, material quality, prototype evaluation, telehealth, home monitoring

## Abstract

**Background/Objectives**: Wearable technologies become increasingly important in surveillance of biometric parameters in pregnant women; however, early-stage usability data on wearable form factors specifically designed for pregnant women remain limited. This study evaluated the usability and acceptance of three non-functional wearable garment prototypes intended for future breathing exercise guidance and sleep-related applications. The prototypes incorporated sensor dummies that were technically capable of operation but intentionally deactivated for this usability pilot study. **Methods**: Eighteen pregnant women (second and third trimester) and twelve non-pregnant women tested three prototypes (Bra, Strap, Maternity Belt (hereafter Belt)) for 24 h. Usability was assessed using structured, participant-completed questionnaires addressing fit, material properties, comfort, and wear-related issues immediately after fitting (T0) and after 24 h of wear (T24). Analyses were descriptive and exploratory. **Results**: Among pregnant women, the Bra prototype showed consistently favorable usability ratings across multiple domains, particularly after extended wear, whereas the Belt demonstrated declining ratings related to fit and comfort over time. The Strap showed intermediate usability with specific strengths related to pressure and friction. In non-pregnant women, usability ratings were largely comparable between the Bra and Strap, with no clear preference pattern. No systematic differences were observed between pregnant and non-pregnant groups. **Conclusions**: This exploratory usability study suggests that garment form factor plays a critical role in acceptability during pregnancy. The Bra prototype demonstrated the most favorable usability profile among pregnant women, while the Belt revealed design limitations that warrant further modification. These findings provide formative guidance for the development of functional maternal wearables, with future studies integrating objective testing and validated measures to optimize performance and evaluate adherence in larger cohorts.

## 1. Introduction

Emerging wearable technologies for pregnant women have the potential to enhance prenatal care by enabling earlier identification of physiological changes, warning signs, and pregnancy-related complications and by supporting preventive strategies [1,2].

Recent systematic reviews of maternal wearables and remote home monitoring indicate that continuous monitoring during pregnancy is increasingly feasible, but implementation barriers remain, particularly user compliance and comfort, data reliability, and data security [2,3]. Sustained adoption depends heavily on end-user comfort and usability. From an engineering perspective, wearable electronics and smart textiles aim to unobtrusively capture physiological signals by integrating flexible sensors into garments while maintaining comfort, washability, and robustness for daily use [4,5]. Before interventions using such garments can be meaningfully implemented, their usability and acceptability in this specific population must be carefully evaluated.

Semi- or non-functional sensor prototypes are evaluated under realistic conditions by participants, with questionnaires used to measure usability factors such as material, comfort, ease of use, and overall acceptance [6]. These insights help establish a solid foundation for a successful, larger-scale feasibility study with the functional device, in which participants remain engaged and the process runs smoothly.

In this pilot study, three wearable prototypes (Bra, Strap, Belt) from Oxa^®^ by Nanoleq (Rumlang, Switzerland) [7], technically capable of operation but intentionally deactivated for the purposes of this usability pilot study and referred to throughout as non-functional prototypes, were tested in pregnant and non-pregnant women. The prototypes were adapted for pregnant women using the existing Oxa^®^ technology for controlled breathing [7].

The objective of this pilot study was to assess the usability and acceptance of pregnancy-related wearable prototypes among pregnant women through structured questionnaires completed immediately after wearable fitting and after 24 h of wear. The study sought to identify the preferred wearable type and potential limitations and user needs associated with the prototypes to inform their validation in a subsequent larger-scale feasibility study of the functional wearable. This study was exploratory and designed to characterize usability and user experience; no formal hypothesis testing was pre-specified. Accordingly, the present study focuses exclusively on garment usability and acceptance as a prerequisite for future functional validation rather than on physiological monitoring or intervention efficacy.

## 2. Materials and Methods

We conducted a monocentric, parallel-group comparative usability pilot study at the University Hospital for Obstetrics and Gynecology in Bern, Switzerland. The study was conducted as part of the international Newlife project, a European collaboration aiming to develop new remote non-invasive monitoring solutions for pregnant women and newborn babies [8].

Participants were assigned to two parallel groups: pregnant women in their second and third trimesters and non-pregnant women.

Inclusion criteria were age ≥18 years and sufficient German language skills to understand study procedures and questionnaires. Exclusion criteria included acute medical conditions (e.g., pain) requiring treatment, known allergies or intolerances to the materials used in the wearables, or skin conditions that could interfere with device use.

**Figure 1:**
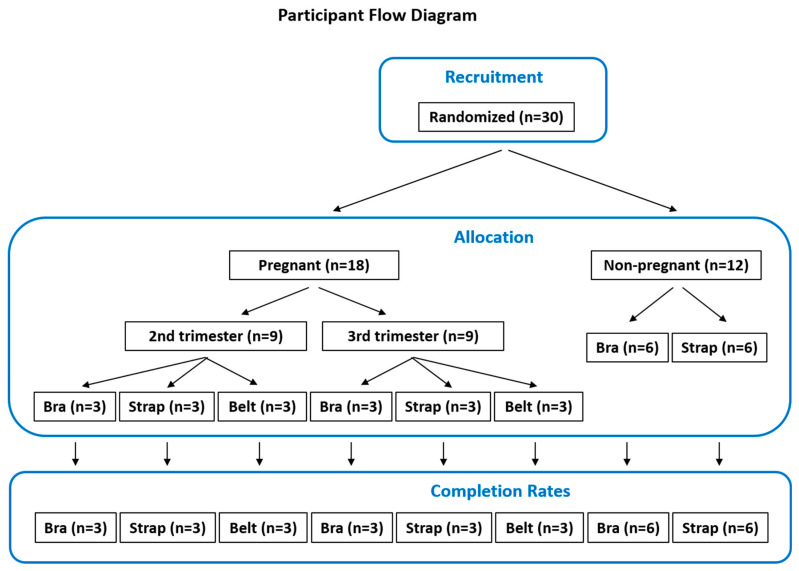
Participant flow diagram.

Recruitment was carried out among patients at the University Hospital for Obstetrics and Gynecology in Bern, Switzerland. Eighteen pregnant women (9 in the second trimester, 9 in the third) and twelve non-pregnant women were recruited for the pregnant and non-pregnant groups, respectively (Figure 1).

The sample size was not based on statistical power calculations, as the primary aim of this study was not hypothesis testing but the identification of usability patterns and potential design limitations. Sample sizes were chosen pragmatically based on feasibility and are consistent with commonly used numbers in early-stage usability testing of wearable devices.

**Figure 2:**
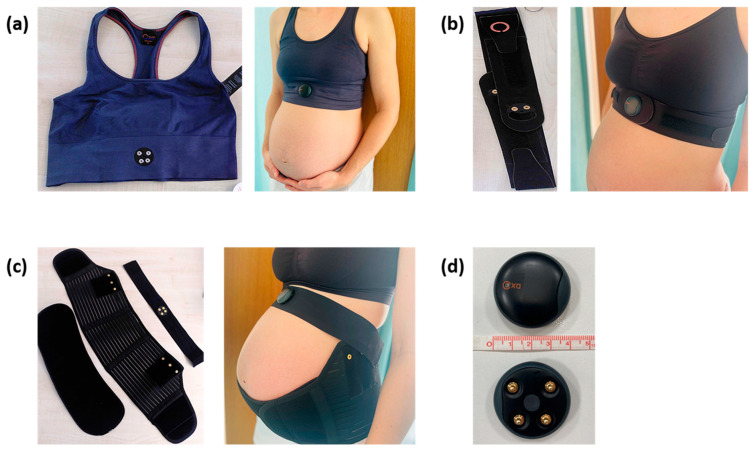
Non-functional wearable prototypes by Oxa^®^ by Nanoleq (Rumlang, Switzerland) modeled and worn by pregnant women: (**a**) Bra, (**b**) Strap, (**c**) Belt, and the (**d**) docking station for sensor placement.

Participants in the pregnant group (*n* = 18) were randomly assigned to one of three wearable types (Bra, Strap, Maternity Belt, hereafter ‘Belt’; see Figure 2) across both trimesters using a computer-based process implemented in SPSS (IBM SPSS Statistics, Version 17; Armonk, NY, USA). Specifically, participants were randomized by generating random numbers. Group allocation was then applied based on rank order to ensure equal distribution. This resulted in three pregnant participants per wearable type per trimester, or six pregnant participants per wearable type across both trimesters. The non-pregnant group (*n* = 12) was randomized to two of the three wearables. The third wearable (Belt) could not be worn by non-pregnant women and was therefore excluded from this group, resulting in six non-pregnant participants per wearable type.

Due to the exploratory nature of the study and the limited sample size, comparisons between pregnant and non-pregnant participants were descriptive. Observed differences between groups, including age imbalance, were considered during interpretation rather than adjusted statistically.

### 2.1. Non-Functional Wearable Prototypes

Three non-functional wearable garment prototypes (Bra, Strap, and Maternity Belt—the latter only for pregnant women) were evaluated, each available in various sizes (Figure 2). These prototypes are intended for future use in breathing exercises.

Each prototype incorporated identical sensor dummies that were technically capable of operation but intentionally deactivated for the purposes of this usability pilot study. The sensor dummies were physically present and attached via a standardized docking station to allow realistic assessment of fit, comfort, and ergonomics without recording physiological data.

The wearables were fitted with the assistance and instruction of a study midwife, and all participants were instructed on independent use. No skin irritation, pressure marks, or other wear-related adverse effects were reported by any participant at either assessment time point.

### 2.2. Measures

Usability was assessed using a study-specific questionnaire developed for this exploratory pilot study. The questionnaire covered multiple usability domains, including fit and size, material properties, comfort and freedom of movement, pressure and friction, ease of use, and overall acceptance.

Different response formats were used to capture domain-specific aspects of usability, including 5-point Likert scales for subjective evaluations, a 3-point semantic differential scale for fit, categorical response options for skin-related issues, and a 10-point numerical rating scale for overall comfort. This multi-scale approach was chosen to reflect the multidimensional nature of usability and to capture nuances not adequately represented by a single composite scale.

The questionnaire was not formally validated, and no reliability metrics (e.g., Cronbach’s alpha) were calculated. Accordingly, results should be interpreted as exploratory. Findings are intended to inform refinement of wearable design and the development of validated measurement tools in subsequent studies.

### 2.3. Data Collection

Participants completed a questionnaire immediately after the initial fitting of the randomly assigned wearable during the instruction by the study midwife (Time point 1: pre-test questionnaire). Participants went home with the wearable for 24 h. After wearing the wearable during both day and night in their daily routine, they completed the questionnaire again (Time point 2: post-test questionnaire).

### 2.4. Ethics Statement

The cantonal ethics committee (CEC) of Bern, Switzerland waived the need for ethics approval for the study (CEC No. 2023-01842). As stated by the CEC, the study qualifies as an opinion survey involving pregnant women, with no collection of health-related data. Moreover, the CEC concluded that the study does not produce generalizable findings and is thus not subject to the provisions of the Human Research Act (Art. 2 and 3 HRA).

The study posed minimal risk, involving short-term use of non-invasive wearable prototypes and questionnaire completion only. No procedures affecting maternal or fetal health were conducted, and all participants provided informed consent after receiving full information about the study and their right to withdraw at any time.

As the study was classified by CEC as being outside the scope of the Human Research Act, registration in a clinical trial registry was neither required nor performed.

## 3. Results

### 3.1. Study Population

Eighteen pregnant women (nine in the second trimester and nine in the third trimester) and twelve non-pregnant women were included. Participant characteristics are summarized in Table 1. Groups were broadly comparable with respect to body size-related parameters (weight, height, BMI, under-bust circumference), while the non-pregnant group was older on average. This age difference is considered in the interpretation of between-group comparisons.

### 3.2. Usability of Wearable Prototypes in Pregnant Women

#### 3.2.1. Initial Usability Impressions (T0)

At initial wear, overall usability ratings were generally favorable for all three prototypes. Clear differences emerged; however, in material- and comfort-related domains (Table 2).

At baseline, the Bra was rated more favorably than the Belt with respect to breathability, perceived sweating, comfort across different temperatures, and suitability for physical activity (sports comfort). The Strap showed intermediate ratings across these domains. No relevant differences were observed between prototypes regarding weight, ease of use, or overall acceptance at baseline (Table 2; complete dataset in Table S1).

#### 3.2.2. Changes After 24 h of Wear (T24)

After 24 h of wear, distinct usability patterns became more pronounced (Table 2). Ratings for the Bra improved across several domains, including fit, material quality, material softness, breathability, everyday comfort, and overall comfort satisfaction. In contrast, ratings for the Belt tended to deteriorate over time, particularly regarding fit, material properties, and overall comfort.

The Strap showed a mixed pattern, with improvements mainly in pressure- and friction-related items, while remaining largely stable in other domains (Table 2 and Table S1).

Overall, after 24 h, the Bra consistently received the most favorable ratings across the majority of usability criteria, whereas the Belt showed the least favorable profile.

#### 3.2.3. Activity-Specific Comfort (Daytime, Sports, Nighttime)

Comfort was assessed separately for different usage contexts, including everyday daytime activities, sports-related movement, and nighttime wear. Activity-specific items are reported in Table 2 and Table S1. This stratification allowed identification of context-dependent usability issues. In pregnant women, the Bra performed consistently well across all contexts, whereas the Belt showed reduced comfort, particularly during prolonged wear and movement-related activities.

### 3.3. Usability of Wearable Prototypes in Non-Pregnant Women

In the non-pregnant group, usability ratings were generally similar between the Bra and the Strap at both assessment time points (Table 3). No consistent preference pattern emerged across most criteria.

The only notable change over time was a reduction in reported skin irritation or allergy potential for both prototypes after 24 h of wear (Table 3 and Table S1). Other usability domains remained largely unchanged.

### 3.4. Comparison Between Pregnant and Non-Pregnant Groups

Comparisons between pregnant and non-pregnant participants did not reveal systematic differences in usability ratings at baseline or after 24 h of wear. Given the exploratory nature of the study and the limited sample size, these findings should be interpreted descriptively.

### 3.5. Summary of Usability Patterns

Taken together, the results indicate a consistent preference for the Bra prototype among pregnant women, particularly after extended wear (Table 4). The Strap demonstrated acceptable usability with specific strengths related to pressure and friction, whereas the Belt showed limitations in comfort and fit that became more apparent over time. Detailed item-level data for all usability criteria are provided in Supplementary Table S1.

## 4. Discussion

To our knowledge, this is the first study to comparatively assess the usability and acceptance of non-functional wearable prototypes intended for maternal health applications in a parallel-group setting. The primary objective was not to evaluate physiological monitoring performance or intervention efficacy, but to identify usability patterns, design limitations, and user preferences among pregnant women in order to inform subsequent development of functional wearable devices.

### 4.1. Key Usability Findings

Across all assessed usability domains, the Bra prototype received consistently the most favorable and stable ratings among pregnant women, both at initial wear and after 24 h of use. In contrast, the Maternity Belt showed declining ratings over time, particularly with respect to fit, material properties, and overall comfort. The Strap prototype consistently showed intermediate usability, with specific lengths related to pressure distribution and friction.

These findings indicate that garment form factor plays a critical role in wearable acceptability during pregnancy, especially under conditions of prolonged wear. Importantly, the observed patterns were consistent across multiple usability domains and usage contexts, including daytime activities, sports-related movement, and nighttime wear.

### 4.2. Interpretation in the Context of Pregnancy Physiology and Ergonomics

Physiological and anatomical changes during pregnancy, including increases in abdominal circumference, breast volume, skin sensitivity, and altered posture, place specific demands on wearable design. Garments that distribute pressure evenly, adapt to changing body contours, and minimize friction are therefore more likely to be tolerated over extended periods [9,10].

The favorable performance of the Bra prototype may be explained by its elastic, close-fitting design and relatively stable positioning on the upper torso, allowing it to accommodate bodily changes while maintaining comfort. In contrast, the Belt prototype, which exerts some pressure on the abdominal region, appears more susceptible to discomfort as pregnancy progresses, particularly during movement and prolonged wear. These findings underscore the importance of pressure distribution, material softness, and adaptability when designing wearable devices for pregnant populations.

### 4.3. Comparison with Other Studies

Usability is a critical factor for the acceptance and effective use of wearable systems, yet it is often under-studied. Dedicated usability evaluation is essential to identify design inconsistencies, improve user satisfaction, and enhance the likelihood that a wearable will be adopted and used in real-world contexts. Specifically, Andreoni highlights that while wearable systems are becoming widespread, their usability, acceptance, and user experience are frequently poorly studied, and structured usability evaluations are necessary to ensure devices work effectively for intended users [6].

While interest in wearable monitoring during pregnancy appears high [2,3,11], only a modest number of studies have conducted formal usability or acceptance evaluations specific to maternal wearables. Existing research includes survey-based assessments of willingness to use health monitoring devices during pregnancy and a small number of usability evaluations of wearable-integrated digital tools, but comprehensive, large-scale usability studies of pregnancy-specific wearables remain limited [12,13]. However, our findings align with those of a previous usability study involving non-pregnant middle-aged women, who expressed a clear preference for upper-torso wearable devices over waist-worn systems [14]. This suggests that device placement and familiarity with specific garment types may substantially influence perceived comfort and acceptability across diverse female populations. The ability to embed sensors in a familiar garment, like a sports bra offers biomechanical stability, distributed pressure, and greater social acceptability than abdomen-centered designs for biometric data collection. The development of smart bras for female health is an emerging area, with applications ranging from heart rate monitoring [15] to early breast cancer detection [16]. However, the technology is still new, and usability studies on smart bras remain limited. A previous study of three commercially available heart rate–sensing smart sports bras during treadmill activity in 24 healthy, non-pregnant women highlights that wearable sports bras must provide both accurate biometric data and comfort, as the most accurate models were not always perceived as the most comfortable [15].

Furthermore, Sarhaddi et al. demonstrated the feasibility of long-term IoT-based (Internet of Things) maternal monitoring using a smartwatch, noting that continuous tracking and energy efficiency are key, while sustaining user engagement remains challenging [17]. This highlights that comfort and user acceptance are crucial for the success of long-term studies.

While prior research has examined functional monitoring systems or telemedicine platforms, direct comparisons to the present study are limited by methodological differences. Notably, the current study focused exclusively on non-functional prototypes to isolate garment-related usability from technical performance. Within this scope, the findings align with existing evidence suggesting that comfort, material quality, and perceived wearability are decisive factors influencing acceptance of wearable technologies in pregnancy [6,10,12,13].

While evaluating garment usability and participant acceptance represents an important initial step, the development of textile-based wearables for real-time biomedical monitoring inherently requires a multidisciplinary approach that integrates textile engineering, materials science, electronics, and data analytics [18]. Thus, the present study covers only a specific aspect of the overall development of a wearable garment. Comprehensive technological system development was not within the scope of this investigation.

### 4.4. Relevance for Future Studies

The insights from this pilot study inform both the further refinement of the wearable prototype and the design of a subsequent clinical study employing functional devices. Based on these findings, we selected the Bra as the most suitable form factor for future studies. In contrast, the Belt prototype requires substantial redesign before further evaluation. Potential improvements include modification of closure mechanisms, redistribution of pressure away from the abdominal region, and the use of more flexible or adaptive materials. The Belt’s decline in comfort over time suggests that abdominally anchored wearables may be particularly sensitive to prolonged pressure and movement-related displacement during pregnancy, necessitating a fundamental redesign rather than incremental refinement.

The Strap prototype demonstrated acceptable usability and may benefit from targeted material refinements to further reduce friction and enhance comfort.

Importantly, the selection of the Bra prototype for further investigation should be understood as a formative design decision rather than a definitive endorsement. The present findings serve to inform the next development phase, which must include functional sensors, objective signal assessment, and evaluation in adequately powered clinical studies.

Future studies have to address wearable biomechanics and garment engineering to demonstrate how movement, pressure distribution, and tissue mechanics influence user experience.

### 4.5. Limitations of the Study

Several limitations must be acknowledged. First, the small sample size precludes definitive conclusions. Accordingly, all findings should be interpreted as exploratory and hypothesis-generating. Second, the questionnaire used to assess usability was study-specific and not formally validated, and no reliability metrics were calculated. Third, the Belt prototype was not tested in non-pregnant participants, limiting full cross-group comparability.

Additionally, an age difference between pregnant and non-pregnant groups may have influenced perceptions of usability. Finally, as the prototypes were non-functional, user experience may differ once functional sensors, additional weight, or real-time feedback are incorporated.

The study did not include objective biomechanical or textile-engineering characterization. However, this pilot was intentionally designed as an early-stage usability and acceptance study using non-functional prototypes, prioritizing participant-reported comfort, fit, and perceived usability as the most relevant outcomes at this development stage. Objective mechanical characterization will be integrated in subsequent iterations to support engineering optimization and clinical validation during functional testing.

## 5. Conclusions and Next Steps

In summary, this exploratory usability study demonstrates that garment form factor substantially influences the acceptability of wearable devices during pregnancy. Among the evaluated prototypes, the Bra showed the most favorable usability profile, while the Belt exhibited clear design limitations.

These findings represent an initial but important step in the development of maternal wearables, providing a foundation for future engineering designs and biomechanical investigations. Future iterations will integrate objective mechanical characterization—such as pressure mapping, strain/tension assessment, and standardized fabric testing—along with functional sensors and validated usability measures to generate actionable engineering parameters and assess performance and adherence in larger, representative cohorts.

## Figures and Tables

**Table 1:** Participants’ characteristics.

	Pregnant Group *n* = 18	Non-Pregnant Group *n* = 12
Age (in years)		
Mean ± SD	32.9 ± 3.8	40.1 ± 8.9
Median (Range)	32 (26–38)	40.5 (27–54)
Weight (in kg)		
Mean ± SD	68.6 ± 10.8	66.9 ± 16
Median (Range)	67 (48–92)	62 (50–109)
Size (in cm)		
Mean ± SD	165.6 ± 5.9	166.5 ± 4.4
Median (Range)	163.5 (159–184)	164 (160–174)
BMI		
Mean ± SD	25.0 ± 4.0	24.0 ± 5.1
Median (Range)	24.4 (19–35)	22.7 (19–38)
Under breast circumference (in cm)		
Mean ± SD	79.8 ± 5.9	80.8 ± 11.4
Median (Range)	81 (68–90)	78.5 (70–105)
Gestational week		Not applicable.
2. Trimester:	*n* = 9	
Mean ± SD	20.2 ± 3.2	
Median (Range)	20 (15–24)	
3. Trimester	*n* = 9	
Mean ± SD	34 ± 3.6	
Median (Range)	36 (28–37)	
Abdominal circumference (in cm)		Not applicable.
Mean ± SD	93.4 ± 8.1	
Median (Range)	95 (77–107)	
Sy-Fu (in cm)		Not applicable.
Mean ± SD	26 ± 7.2	
Median (Range)	24.5 (13–37)	

kg—kilogram, cm—centimeter, BMI—body mass index.

**Table 2:** Evaluation Outcomes in Pregnant Group (Pre/Post-Testing, by Wearable Type).

Criteria (a)–(g)	Item	Wearable	Rating	Pre	Post
				*n*/% N = 6	*n*/% N = 6
(a) Fit and Size	Fit satisfaction	BRA	Very good	2/33.3	3/50.0
			Good	3/50.0	2/33.3
			Partly	1/16.7	1/16.7
			Bad	0/0	0/0
			Very bad	0/0	0/0
		STRAP	Very good	2/33.3	1/16.7
			Good	4/66.7	4/66.7
			Partly	0/0	1/16.7
			Bad	0/0	0/0
			Very bad	0/0	0/0
		BELT	Very good	2/33.3	0/0
			Good	2/33.3	3/50.0
			Partly	2/33.3	0/0
			Bad	0/0	2/33.3
			Very bad	0/0	1/16.7
(b) Materials	Material quality	BRA	Very good	3/50.0	4/66.7
			Good	2/33.3	1/16.7
			Partly	1/16.7	1/16.7
			Bad	0/0	0/0
			Very bad	0/0	0/0
		STRAP	Very good	0/0	0/0
			Good	6/100	6/100
			Partly	0/0	0/0
			Bad	0/0	0/0
			Very bad	0/0	0/0
		BELT	Very good	0/0	1/16.7
			Good	4/66.7	1/16.7
			Partly	2/33.3	1/16.7
			Bad	0/0	2/33.3
			Very bad	0/0	1/16.7
	Material softness	BRA	Very good	3/50.0	4/66.7
			Good	3/50.0	2/33.3
			Partly	0/0	0/0
			Bad	0/0	0/0
			Very bad	0/0	0/0
		STRAP	Very good	0/0	2/33.3
			Good	5/83.3	2/33.3
			Partly	1/16.7	2/33.3
			Bad	0/0	0/0
			Very bad	0/0	0/0
		BELT	Very good	1/16.7	0/0
			Good	3/50.0	2/33.3
			Partly	1/16.7	2/33.3
			Bad	1/16.7	1/16.7
			Very bad	0/0	1/16.7
	Breathability	BRA	Very good	2/33.3	1/16.7
			Good	4/66.7	4/66.7
			Partly	0/0	1/16.7
			Bad	0/0	0/0
			Very bad	0/0	0/0
		STRAP	Very good	0/0	0/0
			Good	2/33.3	3/50.0
			Partly	3/50.0	1/16.7
			Bad	1/16.7	2/33.3
			Very bad	0/0	0/0
		BELT	Very good	0/0	0/0
			Good	2/33.3	1/16.7
			Partly	3/50.0	1/16.7
			Bad	0/0	3/50.0
			Very bad	1/16.7	1/16.7
	Light sweating	BRA	Totally agree	0/0	0/0
			Agree	1/16.7	0/0
			Partly agree	4/66.7	2/33.3
			Tend to disagree	1/16.7	3/50.0
			Do not agree at all	0/0	1/16.7
		STRAP	Totally agree	1/16.7	0/0
			Agree	2/33.3	3/50.0
			Partly agree	3/50.0	1/16.7
			Tend to disagree	0/0	1/16.7
			Do not agree at all	0/0	1/16.7
		BELT	Totally agree	2/33.3	1/16.7
			Agree	3/50.0	2/33.3
			Partly agree	1/16.7	2/33.3
			Tend to disagree	0/0	1/16.7
			Do not agree at all	0/0	0/0
	Comfort at different temperatures	BRA	Totally agree	1/16.7	0/0
			Agree	4/66.7	4/66.7
			Partly agree	1/16.7	2/33.3
			Tend to disagree	0/0	0/0
			Do not agree at all	0/0	0/0
		STRAP	Totally agree	0/0	0/0
			Agree	1/16.7	3/50.0
			Partly agree	5/83.3	3/50.0
			Tend to disagree	0/0	0/0
			Do not agree at all	0/0	0/0
		BELT	Totally agree	0/0	0/0
			Agree	0/0	1/16.7
			Partly agree	2/33.3	3/50.0
			Tend to disagree	4/66.7	1/16.7
			Do not agree at all	0/0	1/16.7
(c) Comfort and Freedom of Movement	Everyday comfort	BRA	Very good	2/33.3	2/33.3
			Good	4/66.7	3/50.0
			Partly	0/0	1/16.7
			Bad	0/0	0/0
			Very bad	0/0	0/0
		STRAP	Very good	0/0	1/16.7
			Good	5/83.3	2/33.3
			Partly	1/16.7	3/50.0
			Bad	0/0	0/0
			Very bad	0/0	0/0
		BELT	Very good	1/16.7	0/0
			Good	3/50.0	2/33.3
			Partly	2/33.3	1/16.7
			Bad	0/0	1/16.7
			Very bad	0/0	2/33.3
	Sports comfort	BRA	Was not worn	0/0	3/50.0
			Very good	2/33.3	1/16.7
			Good	3/50.0	2/33.3
			Partly	1/16.7	0/0
			Bad	0/0	0/0
			Very bad	0/0	0/0
		STRAP	Was not worn	0/0	4/66.7
			Very good	0/0	0/0
			Good	3/50.0	1/16.7
			Partly	3/50.0	0/0
			Bad	0/0	1/16.7
			Very bad	0/0	0/0
		BELT	Was not worn	0/0	4/66.7
			Very good	0/0	0/0
			Good	1/16.7	0/0
			Partly	2/33.3	1/16.7
			Bad	3/50.0	0/0
			Very bad	0/0	1/16.7
(g) Overall acceptance	Overall comfort satisfaction	BRA	Very satisfied	2/33.3	1/16.7
			Satisfied	4/66.7	4/66.7
			Partly	0/0	1/16.7
			Rather dissatisfied	0/0	0/0
			Not at all satisfied	0/0	0/0
		STRAP	Very satisfied	1/16.7	0/0
			Satisfied	5/83.3	4/66.7
			Partly	0/0	2/33.3
			Rather dissatisfied	0/0	0/0
			Not at all satisfied	0/0	0/0
		BELT	Very satisfied	1/16.7	0/0
			Satisfied	3/50.0	1/16.7
			Partly	1/16.7	2/33.3
			Rather dissatisfied	1/16.7	1/16.7
			Not at all satisfied	0/0	2/33.3

Pre—Assessment at initial wearing; Post—Assessment after 24 h of wearing.

**Table 3:** Evaluation Outcomes in Non-Pregnant Group (Pre/Post-Testing, by Wearable Type).

Criteria (a)–(g)	Item	Wearable	Rating	Pre	Post
				*n*/% N = 6	*n*/% N = 6
(a) Fit and Size	Fit satisfaction	BRA	Very good	2/33.3	2/33.3
			Good	4/66.7	3/50.0
			Partly	0/0	1/16.7
			Bad	0/0	0/0
			Very bad	0/0	0/0
		STRAP	Very good	2/33.3	2/33.3
			Good	3/50.0	3/50.0
			Partly	1/16.7	1/16.7
			Bad	0/0	0/0
			Very bad	0/0	0/0
(b) Materials	Material quality	BRA	Very good	4/66.7	4/66.7
			Good	2/33.3	2/33.3
			Partly	0/0	0/0
			Bad	0/0	0/0
			Very bad	0/0	0/0
		STRAP	Very good	1/16.7	2/33.3
			Good	3/50.0	4/66.7
			Partly	2/33.3	0/0
			Bad	0/0	0/0
			Very bad	0/0	0/0
	Material softness	BRA	Very good	5/83.3	4/66.7
			Good	1/16.7	2/33.3
			Partly	0/0	0/0
			Bad	0/0	0/0
			Very bad	0/0	0/0
		STRAP		(*n* = 5)	
			Very good	2/40.0	2/33.3
			Good	2/40.0	4/66.7
			Partly	1/20.0	0/0
			Bad	0/0	0/0
			Very bad	0/0	0/0
	Breathability	BRA	Very good	2/33.3	2/33.3
			Good	4/66.7	3/50.0
			Partly	0/0	0/0
			Bad	0/0	1/16.7
			Very bad	0/0	0/0
		STRAP	Very good	1/16.7	1/16.7
			Good	2/33.3	2/33.3
			Partly	3/50.0	2/33.3
			Bad	0/0	1/16.7
			Very bad	0/0	0/0
	Light sweating	BRA	Totally agree	1/16.7	0/0
			Agree	0/0	1/16.7
			Partly agree	3/50.0	2/33.3
			Tend to disagree	2/33.3	3/50.0
			Do not agree at all	0/0	0/0
		STRAP	Totally agree	0/0	1/16.7
			Agree	3/50.0	2/33.3
			Partly agree	2/33.3	0/0
			Tend to disagree	1/16.7	1/16.7
			Do not agree at all	0/0	2/33.3
	Comfort at different temperatures	BRA	Totally agree	0/0	0/0
			Agree	2/33.3	2/33.3
			Partly agree	4/66.7	4/66.7
			Tend to disagree	0/0	0/0
			Do not agree at all	0/0	0/0
		STRAP	Totally agree	2/33.3	2/33.3
			Agree	1/16.7	1/16.7
			Partly agree	3/50.0	3/50.0
			Tend to disagree	0/0	0/0
			Do not agree at all	0/0	0/0
(c) Comfort and Freedom of Movement	Everyday comfort	BRA	Very good	2/33.3	2/33.3
			Good	4/66.7	3/50.0
			Partly	0/0	1/16.7
			Bad	0/0	0/0
			Very bad	0/0	0/0
		STRAP	Very good	1/16.7	1/16.7
			Good	5/83.3	3/50.0
			Partly	0/0	2/33.3
			Bad	0/0	0/0
			Very bad	0/0	0/0
	Sports comfort	BRA	Was not worn	0/0	6/100
			Very good	3/50.0	0/0
			Good	0/0	0/0
			Partly	2/33.3	0/0
			Bad	1/16.7	0/0
			Very bad	0/0	0/0
		STRAP	Was not worn	0/0	3/50.0
			Very good	1/16.7	1/16.7
			Good	1/16.7	0/0
			Partly	3/50.0	1/16.7
			Bad	1/16.7	1/16.7
			Very bad	0/0	0/0
(g) Overall acceptance	Overall comfort satisfaction	BRA	Very satisfied	2/33.3	2/33.3
			Satisfied	2/33.3	3/50.0
			Partly	2/33.3	1/16.7
			Rather dissatisfied	0/0	0/0
			Not at all satisfied	0/0	0/0
		STRAP	Very satisfied	2/33.3	3/50.0
			Satisfied	4/66.7	1/16.7
			Partly	0/0	2/33.3
			Rather dissatisfied	0/0	0/0
			Not at all satisfied	0/0	0/0

Pre—Assessment at initial wearing; Post—Assessment after 24 h of wearing.

**Table 4:** Wearable Preferences by Pregnant and Non-Pregnant Groups.

	Pregnant Group						Non-Pregnant Group			
	Pre			Post			Pre		Post	
Criteria	Bra	Strap	Belt	Bra	Strap	Belt	Bra	Strap	Bra	Strap
(a) Fit and size		X		X			*	*	X	
(b) Materials	X			X			X		X	
(c) Comfort and Freedom of Movement	X			X			X		X	
(d) Pressure Points, Friction, and Allergies	X			*	*		*	*	*	*
(e) Weight		X		X				X		X
(f) Usability		X		X			X		*	*
(g) Overall acceptance	X			X				X		X

X—indicates the preference for the wearable within each criterion by the pregnant or non-pregnant group; *—denotes a tie or no preference; Pre—Assessment at the initial wearing; Post—Assessment after 24 h of wearing.

## Data Availability

The entire dataset is provided in Supporting Information Table S1. Personal data are not publicly available due to ethical and legal restrictions.

## Supplementary Materials

The following supporting information can be downloaded at: https://www.mdpi.com/article/10.3390/healthcare14050618/s1, Table S1: In-depth overview of the complete dataset, covering all criteria and items for both the pregnant and non-pregnant groups (DOCX).

## Abbreviations

The following abbreviations are used in this manuscript:

CEC	Cantonal Ethics Committee
HRA	Human Research Act
