# Supplementary material for: Usability and Acceptance of Non-Functional Wearable Prototypes for Maternal Health: A Parallel-Group Pilot Study
Source: Healthcare (Basel). 2026 Feb 28;14(5):618. doi: 10.3390/healthcare14050618 (PMC12984298; doi:10.3390/healthcare14050618)
Supplement: Supplementary file 1 [file healthcare-14-00618-s001.zip › healthcare-4109812-supplementary.pdf]

Usability and Acceptance of Non-Functional Wearable Proto-types for Maternal Health: A Parallel-Group Pilot Study

Julia Jockusch, Sophie Schneider, Andrea Hochuli, Flurin Stauffer, Heike Bördgen, Vanessa Hoop, Marianne Joerger-Messerli, Daniel Surbek, Anda-Petronela Radan

Table S1. **In-depth overview of the complete dataset, covering all criteria and items for both the Pregnant and Non-Pregnant group.** Evaluation results are separated by pre- and post-usability testing and wearable type (Bra, Strap, Belt). Pre - Assessment at the initial wearing; Post - Assessment after 24 hours of wearing.

			**Pregnant Group**		**Non-Pregnant Group**	
**Criteria (a)-(g) and Items**	**Wearable**	**Rating**	**Pre**	**Post**	**Pre**	**Post**
**(a) Fit and size**						
Item: Fit satisfaction<br>Question: How would you rate the fit of the wearable at the time of assessment? (n/%)	BRA	Very good	2 / 33.3	3 / 50.0	2 / 33.3	2 / 33.3
		Good	3 / 50.0	2 / 33.3	4 / 66.7	3 / 50.0
		Partly	1 / 16.7	1 / 16.7	0 / 0	1 / 16.7
		Bad	0 / 0	0 / 0	0 / 0	0 / 0
		Very bad	0 / 0	0 / 0	0 / 0	0 / 0
	STRAP	Very good	2 / 33.3	1 / 16.7	2 / 33.3	2 / 33.3
		Good	4 / 66.7	4 / 66.7	3 / 50.0	3 / 50.0
		Partly	0 / 0	1 / 16.7	1 / 16.7	1 / 16.7
		Bad	0 / 0	0 / 0	0 / 0	0 / 0
		Very bad	0 / 0	0 / 0	0 / 0	0 /0
	BELT	Very good	2 / 33.3	0 / 0	Not applicable	
		Good	2 / 33.3	3 / 50.0		
		Partly	2 / 33.3	0 / 0		
		Bad	0 / 0	2 / 33.3		
		Very bad	0 / 0	1 / 16.7		
Item: Size satisfaction<br>Question: How does the fit of the wearable feel: too tight, too loose, or just right? (n/%)	BRA	Too tight	1 / 16.7	1 / 16.7	2 / 33.3	2 / 33.3
		Too loose	0 / 0	0 / 0	0 / 0	1 / 16.7
		Just right	5 / 83.3	5 / 83.3	4 / 66.7	3 / 50.0
	STRAP	Too tight	0 / 0	1 / 16.7	0 / 0	1 / 16.7
		Too loose	0 / 0	1 / 16.7	0 / 0	1 / 16.7
		Just right	6 / 100	4 / 66.7	6 / 100	4 / 66.7
	BELT	Too tight	0 / 0	2 / 33.3	Not applicable	
		Too loose	0 / 0	1 / 16.7		
		Just right	5 / 100	3 / 50.0		

Table S1. (continued)

**(b) Materials**						
Item:<br>Material quality<br>Question:<br>How would you rate the quality of the materials used in the wearable?<br>(n/%)	BRA	Very good	3 / 50.0	4 / 66.7	4 / 66.7	4 / 66.7
		Good	2 / 33.3	1 / 16.7	2 / 33.3	2 / 33.3
		Partly	1 / 16.7	1 / 16.7	0 / 0	0 / 0
		Bad	0 / 0	0 / 0	0 / 0	0 / 0
		Very bad	0 / 0	0 / 0	0 / 0	0 / 0
	STRAP	Very good	0 / 0	0 / 0	1 / 16.7	2 / 33.3
		Good	6 / 100	6 / 100	3 / 50.0	4 / 66.7
		Partly	0 / 0	0 / 0	2 / 33.3	0 / 0
		Bad	0 / 0	0 / 0	0 / 0	0 / 0
		Very bad	0 / 0	0 / 0	0 / 0	0 / 0
	BELT	Very good	0 / 0	1 / 16.7	Not applicable	
		Good	4 / 66.7	1 / 16.7		
		Partly	2 / 33.3	1 / 16.7		
		Bad	0 / 0	2 / 33.3		
		Very bad	0 / 0	1 / 16.7		
Item:<br>Material softness<br>Question:<br>How would you rate the softness of the materials used in the wearable?<br>(n/%)	BRA	Very good	3 / 50.0	4 / 66.7	5 / 83.3	4 / 66.7
		Good	3 / 50.0	2 / 33.3	1 / 16.7	2 / 33.3
		Partly	0 / 0	0 / 0	0 / 0	0 / 0
		Bad	0 / 0	0 / 0	0 / 0	0 / 0
		Very bad	0 / 0	0 / 0	0 / 0	0 / 0
	STRAP				(n=5)	
		Very good	0 / 0	2 / 33.3	2 / 40.0	2 / 33.3
		Good	5 / 83.3	2 / 33.3	2 / 40.0	4 / 66.7
		Partly	1 / 16.7	2 / 33.3	1 / 20.0	0 / 0
		Bad	0 / 0	0 / 0	0 / 0	0 / 0
		Very bad	0 / 0	0 / 0	0 / 0	0 / 0
	BELT	Very good	1 / 16.7	0 / 0	Not applicable	
		Good	3 / 50.0	2 / 33.3		
		Partly	1 / 16.7	2 / 33.3		
		Bad	1 / 16.7	1 / 16.7		
		Very bad	0 / 0	1 / 16.7		

Usability and Acceptance of Non-Functional Wearable Proto-types for Maternal Health: A Parallel-Group Pilot Study

Julia Jockusch, Sophie Schneider, Andrea Hochuli, Flurin Stauffer, Heike Bördgen, Vanessa Hoop, Marianne Joerger-Messerli, Daniel Surbek, Anda-Petronela Radan

Table S1. (continued)

**(b) Materials**						
Item: Material comfort<br>Question: ow would you rate the overall comfort of the materials used in the wearable? (n/%)	BRA	Very good	2 / 33.3	4 / 66.7	4 / 66.7	4 / 66.7
		Good	4 / 66.7	1 / 16.7	1 / 16.7	1 / 16.7
		Partly	0 / 0	1 / 16.7	1 / 16.7	1 / 16.7
		Bad	0 / 0	0 / 0	0 / 0	0 / 0
		Very bad	0 / 0	0 / 0	0 / 0	0 / 0
	STRAP	Very good	1 / 16.7	1 / 16.7	1 / 16.7	1 / 16.7
		Good	5 / 83.3	2 / 33.3	4 / 66.7	4 / 66.7
		Partly	0 / 0	3 / 50.0	1 / 16.7	1 / 16.7
		Bad	0 / 0	0 / 0	0 / 0	0 / 0
		Very bad	0 / 0	0 / 0	0 / 0	0 / 0
	BELT	Very good	1 / 16.7	0 / 0	Not applicable	
		Good	4 / 66.7	3 / 50.0		
		Partly	0 / 0	1 / 16.7		
		Bad	1 / 16.7	1 / 16.7		
		Very bad	0 / 0	1 / 16.7		
Item: Breathability<br>Question: Do you find the wearable to be breathable? (n/%)	BRA	Totally agree	2 / 33.3	1 / 16.7	2 / 33.3	2 / 33.3
		Agree	4 / 66.7	4 / 66.7	4 / 66.7	3 / 50.0
		Partly agree	0 / 0	1 / 16.7	0 / 0	0 / 0
		Tend to disagree	0 / 0	0 / 0	0 / 0	1 / 16.7
		Do not agree at all	0 / 0	0 / 0	0 / 0	0 / 0
	STRAP	Totally agree	0 / 0	0 / 0	1 / 16.7	1 / 16.7
		Agree	2 / 33.3	3 / 50.0	2 / 33.3	2 / 33.3
		Partly agree	3 / 50.0	1 / 16.7	3 / 50.0	2 / 33.3
		Tend to disagree	1 / 16.7	2 / 33.3	0 / 0	1 / 16.7
		Do not agree at all	0 / 0	0 / 0	0 / 0	0 / 0
	BELT	Totally agree	0 / 0	0 / 0	Not applicable	
		Agree	2 / 33.3	1 / 16.7		
		Partly agree	3 / 50.0	1 / 16.7		
		Tend to disagree	0 / 0	3 / 50.0		
		Do not agree at all	1 / 16.7	1 / 16.7		

Usability and Acceptance of Non-Functional Wearable Proto-types for Maternal Health: A Parallel-Group Pilot Study

Julia Jockusch, Sophie Schneider, Andrea Hochuli, Flurin Stauffer, Heike Bördgen, Vanessa Hoop, Marianne Joerger-Messerli, Daniel Surbek, Anda-Petronela Radan

Table S1. (continued)

**(b) Materials**						
Item: Light sweating Question: Do you feel that you might experience light sweating while wearing the wearable? (n/%)	BRA	Totally agree	0 / 0	0 / 0	1 / 16.7	0 / 0
		Agree	1 / 16.7	0 / 0	0 / 0	1 / 16.7
		Partly agree	4 / 66.7	2 / 33.3	3 / 50.0	2 / 33.3
		Tend to disagree	1 / 16.7	3 / 50.0	2 / 33.3	3 / 50.0
		Do not agree at all	0 / 0	1 / 16.7	0 / 0	0 / 0
	STRAP	Totally agree	1 / 16.7	0 / 0	0 / 0	1 / 16.7
		Agree	2 / 33.3	3 / 50.0	3 / 50.0	2 / 33.3
		Partly agree	3 / 50.0	1 / 16.7	2 / 33.3	0 / 0
		Tend to disagree	0 / 0	1 / 16.7	1 / 16.7	1 / 16.7
		Do not agree at all	0 / 0	1 / 16.7	0 / 0	2 / 33.3
	BELT	Totally agree	2 / 33.3	1 / 16.7	Not applicable	
		Agree	3 / 50.0	2 / 33.3		
		Partly agree	1 / 16.7	2 / 33.3		
		Tend to disagree	0 / 0	1 / 16.7		
		Do not agree at all	0 / 0	0 / 0		
Item: Comfort at different temperatures Question: Do you think you would feel comfortable wearing the wearable at different temperatures? (n/%)	BRA	Totally agree	1 / 16.7	0 / 0	0 / 0	0 / 0
		Agree	4 / 66.7	4 /66.7	2 / 33.3	2 / 33.3
		Partly agree	1 / 16.7	2 / 33.3	4 / 66.7	4 / 66.7
		Tend to disagree	0 / 0	0 / 0	0 / 0	0 / 0
		Do not agree at all	0 / 0	0 / 0	0 / 0	0 / 0
	STRAP	Totally agree	0 / 0	0 / 0	2 / 33.3	2 / 33.3
		Agree	1 / 16.7	3 / 50.0	1 / 16.7	1 / 16.7
		Partly agree	5 / 83.3	3 / 50.0	3 / 50.0	3 / 50.0
		Tend to disagree	0 / 0	0 / 0	0 / 0	0 / 0
		Do not agree at all	0 / 0	0 / 0	0 / 0	0 / 0
	BELT	Totally agree	0 / 0	0 / 0	Not applicable	
		Agree	0 / 0	1 / 16.7		
		Partly agree	2 / 33.3	3 / 50.0		
		Tend to disagree	4 / 66.7	1 / 16.7		
		Do not agree at all	0 / 0	1 / 16.7		

Usability and Acceptance of Non-Functional Wearable Proto-types for Maternal Health: A Parallel-Group Pilot Study

Julia Jockusch, Sophie Schneider, Andrea Hochuli, Flurin Stauffer, Heike Bördgen, Vanessa Hoop, Marianne Joerger-Messerli, Daniel Surbek, Anda-Petronela Radan

Table S1. (continued)

**(c) Comfort and Freedom of Movement**						
Item: Movement restriction. Question: Do you feel that your movements are restricted by the wearable? (n/%)	BRA	Totally agree	0 / 0	0 / 0	0 / 0	0 / 0
		Agree	0 / 0	2 / 33.3	0 / 0	0 / 0
		Partly agree	0 / 0	0 / 0	1 / 16.7	0 / 0
		Tend to disagree	5 / 83.3	1 / 16.7	3 / 50.0	4 / 66.7
		Do not agree at all	1 / 16.7	3 / 50.0	2 / 33.3	2 / 33.3
	STRAP	Totally agree	0 / 0	0 / 0	0 / 0	0 / 0
		Agree	0 / 0	0 / 0	1 / 16.7	0 / 0
		Partly agree	0 / 0	1 / 16.7	1 / 16.7	1 / 16.7
		Tend to disagree	5 / 83.3	3 / 50.0	1 / 16.7	3 / 50.0
		Do not agree at all	1 / 16.7	2 / 33.3	3 / 50.0	2 / 33.3
	BELT	Totally agree	0 / 0	1 / 16.7	Not applicable	
		Agree	1 / 16.7	1 / 16.7		
		Partly agree	2 / 33.3	1 / 16.7		
		Tend to disagree	2 / 33.3	0 / 0		
		Do not agree at all	1 / 16.7	3 / 50.0		
Item: Everyday comfort. Question: How would you rate the comfort of the wearable during daily activities? (n/%)	BRA	Very good	2 / 33.3	2 / 33.3	2 / 33.3	2 / 33.3
		Good	4 / 66.7	3 / 50.0	4 / 66.7	3 / 50.0
		Partly	0 / 0	1 / 16.7	0 / 0	1 / 16.7
		Bad	0 / 0	0 / 0	0 / 0	0 / 0
		Very bad	0 / 0	0 / 0	0 / 0	0 / 0
	STRAP	Very good	0 / 0	1 / 16.7	1 / 16.7	1 / 16.7
		Good	5 / 83.3	2 / 33.3	5 / 83.3	3 / 50.0
		Partly	1 / 16.7	3 / 50.0	0 / 0	2 / 33.3
		Bad	0 / 0	0 / 0	0 / 0	0 / 0
		Very bad	0 / 0	0 / 0	0 / 0	0 / 0
	BELT	Very good	1 / 16.7	0 / 0	Not applicable	
		Good	3 / 50.0	2 / 33.3		
		Partly	2 / 33.3	1 / 16.7		
		Bad	0 / 0	1 / 16.7		
		Very bad	0 / 0	2 / 33.3		

Table S1. (continued)

**(c) Comfort and Freedom of Movement**						
Item:<br>Sports comfort<br>Question:<br>How would you rate the comfort of the wearable during physical activities or sports?<br>(n/%)	BRA	Was not worn	0 / 0	3 / 50.0	0 / 0	6 / 100
		Very good	2 / 33.3	1 / 16.7	3 / 50.0	0 / 0
		Good	3 / 50.0	2 / 33.3	0 / 0	0 / 0
		Partly	1 / 16.7	0 / 0	2 / 33.3	0 / 0
		Bad	0 / 0	0 / 0	1 / 16.7	0 / 0
		Very bad	0 / 0	0 / 0	0 / 0	0 / 0
	STRAP	Was not worn	0 / 0	4 / 66.7	0 / 0	3 / 50.0
		Very good	0 / 0	0 / 0	1 / 16.7	1 / 16.7
		Good	3 / 50.0	1 / 16.7	1 / 16.7	0 / 0
		Partly	3 / 50.0	0 / 0	3 / 50.0	1 / 16.7
		Bad	0 / 0	1 / 16.7	1 / 16.7	1 / 16.7
		Very bad	0 / 0	0 / 0	0 / 0	0 / 0
	BELT	Was not worn	0 / 0	4 /66.7	Not applicable	
		Very good	0 / 0	0 / 0		
		Good	1 / 16.7	0 / 0		
		Partly	2 / 33.3	1 / 16.7		
		Bad	3 / 50.0	0 / 0		
		Very bad	0 / 0	1 / 16.7		
Item:<br>Nighttime comfort<br>Question:<br>How would you rate the comfort of the wearable during the night or while sleeping?<br>(n/%)	BRA	Was not worn	0 / 0	2 / 33.3	0 / 0	0 / 0
		Very good	0 / 0	0 / 0	0 / 0	1 / 16.7
		Good	2 / 33.3	1 / 16.7	4 / 66.7	4 / 66.7
		Partly	4 / 66.7	0 / 0	2 / 33.3	1 / 16.7
		Bad	0 / 0	2 / 33.3	0 / 0	0 / 0
		Very bad	0 / 0	1 / 16.7	0 / 0	0 / 0
	STRAP	Was not worn	0 / 0	0 / 0	0 / 0	1 / 16.7
		Very good	0 / 0	0 / 0	1 / 16.7	2 / 33.3
		Good	2 / 33.3	3 / 50.0	2 / 33.3	1 / 16.7
		Partly	3 / 50.0	2 / 33.3	2 / 33.3	1 / 16.7
		Bad	1 / 16.7	1 / 16.7	1 / 16.7	1 / 16.7
		Very bad	0 / 0	0 / 0	0 / 0	0 / 0
	BELT	Was not worn	0 / 0	2 / 33.3	Not applicable	
		Very good	0 / 0	0 / 0		
		Good	2 / 33.3	1 / 16.7		
		Partly	2 / 33.3	0 / 0		
		Bad	2 / 33.3	2 / 33.3		
		Very bad	0 / 0	1 / 16.7		

Usability and Acceptance of Non-Functional Wearable Proto-types for Maternal Health: A Parallel-Group Pilot Study

Julia Jockusch, Sophie Schneider, Andrea Hochuli, Flurin Stauffer, Heike Bördgen, Vanessa Hoop, Marianne Joerger-Messerli, Daniel Surbek, Anda-Petronela Radan

Table S1. (continued)

**(d) Pressure Points, Friction, and Allergies**						
<u>Item:</u><br>Pressure Points and Friction<br><u>Question:</u><br>Do you feel that the wearable might cause pressure points or friction on your skin?<br>(n/%)	BRA	No	3 / 50.0	3 / 50.0	1 / 16.7	4 / 66.7
		Yes	0 / 0	3 / 50.0	1 / 16.7	2 / 33.3
		Maybe	3 / 50.0	0 / 0	4 / 66.7	0 / 0
		Do not know	0 / 0	0 / 0	0 / 0	0 / 0
	STRAP	No	2 / 33.3	5 / 83.3	4 / 66.7	4 / 66.7
		Yes	1 / 16.7	1 / 16.7	0 / 0	1 / 16.7
		Maybe	3 / 50.0	0 / 0	2 / 33.3	1 / 16.7
		Do not know	0 / 0	0 / 0	0 / 0	0 / 0
	BELT	No	2 / 33.3	2 / 33.3	Not applicable	
		Yes	1 / 16.7	4 / 66.7		
		Maybe	3 / 50.0	0 / 0		
		Do not know	0 / 0	0 / 0		
<u>Item:</u><br>Skin irritations and allergies<br><u>Question:</u><br>Do you think that using the wearable might cause skin irritations or allergies?<br>(n/%)	BRA	Very likely	0 / 0	0 / 0	0 / 0	0 / 0
		Probably	0 / 0	0 / 0	1 / 16.7	0 / 0
		Partly	0 / 0	0 / 0	1 / 16.7	0 / 0
		Rather unlikely	5 /83.3	0 / 0	4 / 66.7	1/ 16.7
		No	1 / 16.7	6 / 100	0 / 0	5 / 83.3
	STRAP	Very likely	0 / 0	0 / 0	0 / 0	0 / 0
		Probably	1 / 16.7	0 / 0	2 / 33.3	0 / 0
		Partly	1 / 16.7	1 / 16.7	1 / 16.7	2 / 33.3
		Rather unlikely	4 / 66.7	0 / 0	3 / 50.0	0 / 0
		No	0 / 0	5 / 83.3	0 / 0	4 / 66.7
	BELT	Very likely	0 / 0	1 / 16.7	Not applicable	
		Probably	0 / 0	0 / 0		
		Partly	4 / 66.7	0 / 0		
		Rather unlikely	2 / 33.3	1 / 16.7		
		No	0 / 0	4 / 66.7		

Usability and Acceptance of Non-Functional Wearable Proto-types for Maternal Health: A Parallel-Group Pilot Study

Julia Jockusch, Sophie Schneider, Andrea Hochuli, Flurin Stauffer, Heike Bördgen, Vanessa Hoop, Marianne Joerger-Messerli, Daniel Surbek, Anda-Petronela Radan

Table S1. (continued)

**(e) Weight**						
Item:<br>Weight appropriate-ness<br>Question:<br>Do you find the weight of the wearable to be appropriate? (n/%)	BRA	Totally agree	2 / 33.3	4 / 66.7	3 / 50.0	3 / 50.0
		Agree	4 / 66.7	2 / 33.3	2 / 33.3	3 / 50.0
		Partly agree	0 / 0	0 / 0	1 / 16.7	0 / 0
		Tend to disagree	0 / 0	0 / 0	0 / 0	0 / 0
		Do not agree at all	0 / 0	0 / 0	0 / 0	0 / 0
	STRAP	Totally agree	3 / 50.0	2 / 33.3	4 / 66.7	4 / 66.7
		Agree	3 / 50.0	3 / 50.0	2 / 33.3	1 / 16.7
		Partly agree	0 / 0	1 / 16.7	0 / 0	1 / 16.7
		Tend to disagree	0 / 0	0 / 0	0 / 0	0 / 0
		Do not agree at all	0 / 0	0 / 0	0 / 0	0 / 0
	BELT	Totally agree	2 / 33.3	1 / 16.7		
		Agree	4 / 66.7	3 / 50.0		
		Partly agree	0 / 0	1 / 16.7	Not applicable	
		Tend to disagree	0 / 0	0 / 0		
		Do not agree at all	0 / 0	1 / 16.7		
**(f) Usability**						
Item:<br>Usability<br>Question:<br>Is putting on the wearable easy and user-friendly?<br>(n/%)	BRA	Totally agree	3 / 50.0	4 / 66.7	4 / 66.7	3 / 50.0
		Agree	2 / 33.3	2 / 33.3	2 / 33.3	3 / 50.0
		Partly agree	0 / 0	0 / 0	0 / 0	0 / 0
		Tend to disagree	1 / 16.7	0 / 0	0 / 0	0 / 0
		Do not agree at all	0 / 0	0 / 0	0 / 0	0 / 0
	STRAP	Totally agree	3 / 50.0	2 / 33.3	2 / 33.3	3 / 50.0
		Agree	3 / 50.0	3 / 50.0	3 / 50.0	3 / 50.0
		Partly agree	0 / 0	1 / 16.7	1 / 16.7	0 / 0
		Tend to disagree	0 / 0	0 / 0	0 / 0	0 / 0
		Do not agree at all	0 / 0	0 / 0	0 / 0	0 / 0
	BELT	Totally agree	1 / 16.7	1 / 16.7		
		Agree	4 / 66.7	4 / 66.7		
		Partly agree	1 / 16.7	0 / 0	Not applicable	
		Tend to disagree	0 / 0	1 / 16.7		
		Do not agree at all	0 / 0	0 / 0		

Usability and Acceptance of Non-Functional Wearable Proto-types for Maternal Health: A Parallel-Group Pilot Study

Julia Jockusch, Sophie Schneider, Andrea Hochuli, Flurin Stauffer, Heike Bördgen, Vanessa Hoop, Marianne Joerger-Messerli, Daniel Surbek, Anda-Petronela Radan

Table S1. (continued)

**(g) Overall accpetance**						
Item:<br>Overall comfort satisfaction<br>Question:<br>How satisfied are you overall with the comfort of the wearable?<br>(n/%)	BRA	Very satisfied<br>Satisfied<br>Partly<br>Rather dissatisfied<br>Not at all satisfied	2 / 33.3<br>4 / 66.7<br>0 / 0<br>0 / 0<br>0 / 0	1 / 16.7<br>4 / 66.7<br>1 / 16.7<br>0 / 0<br>0 / 0	2 / 33.3<br>2 / 33.3<br>2 / 33.3<br>0 / 0<br>0 / 0	2 / 33.3<br>3 / 50.0<br>1 / 16.7<br>0 / 0<br>0 / 0
	STRAP	Very satisfied<br>Satisfied<br>Partly<br>Rather dissatisfied<br>Not at all satisfied	1 / 16.7<br>5 / 83.3<br>0 / 0<br>0 / 0<br>0 / 0	0 / 0<br>4 / 66.7<br>2 / 33.3<br>0 / 0<br>0 / 0	2 / 33.3<br>4 / 66.7<br>0 / 0<br>0 / 0<br>0 / 0	3 / 50.0<br>1 / 16.7<br>2 / 33.3<br>0 / 0<br>0 / 0
	BELT	Very satisfied<br>Satisfied<br>Partly<br>Rather dissatisfied<br>Not at all satisfied	1 / 16.7<br>3 / 50.0<br>1 / 16.7<br>1 / 16.7<br>0 / 0	0 / 0<br>1 / 16.7<br>2 / 33.3<br>1 / 16.7<br>2 / 33.3	Not applicable	
Item:<br>Overall comfort rating<br>Question:<br>How would you rate the overall comfort of the wearable on a scale from 1 to 10?	BRA	Mean ± SD<br>Median (Range)	8 ± 1.7<br>8.5 (6-10)	7.8 ± 1.5<br>8 (5-9)	7.2 ± 2.1<br>7 (5-10)	7.8 ± 1.3<br>8 (6-9)
	STRAP	Mean ± SD<br>Median (Range)	7.5 ± 1.8<br>7 (5-10)	7 ± 1.7<br>7 (4-9)	8.2 ± 1.8<br>8 (6-10)	8.3 ± 1.2<br>8.5 (7-10)
	BELT	Mean ± SD<br>Median (Range)	6.7 ± 1.9<br>7 (4-9)	4.7 ± 3.1<br>5.5 (0-8)	Not applicable	
